# Imaging of Disease Dynamics during Meningococcal Sepsis

**DOI:** 10.1371/journal.pone.0000241

**Published:** 2007-02-21

**Authors:** Hong Sjölinder, Ann-Beth Jonsson

**Affiliations:** Department of Medical Biochemistry and Microbiology, Biomedical Center, Uppsala University, Uppsala, Sweden; Brighton and Sussex Medical School, United Kingdom

## Abstract

*Neisseria meningitidis* is a human pathogen that causes septicemia and meningitis with high mortality. The disease progression is rapid and much remains unknown about the disease process. The understanding of disease development is crucial for development of novel therapeutic strategies and vaccines against meningococcal disease. The use of bioluminescent imaging combined with a mouse disease model allowed us to investigate the progression of meningococcal sepsis over time. Injection of bacteria in blood demonstrated waves of bacterial clearance and growth, which selected for Opa-expressing bacteria, indicating the importance of this bacterial protein. Further, *N. meningitidis* accumulated in the thyroid gland, while thyroid hormone T4 levels decreased. Bacteria reached the mucosal surfaces of the upper respiratory tract, which required expression of the meningococcal PilC1 adhesin. Surprisingly, PilC1 was dispensable for meningococcal growth in blood and for crossing of the blood-brain barrier, indicating that the major role of PilC1 is to interact with mucosal surfaces. This *in vivo* study reveals disease dynamics and organ targeting during meningococcal disease and presents a potent tool for further investigations of meningococcal pathogenesis and vaccines *in vivo*. This might lead to development of new strategies to improve the outcome of meningococcal disease in human patients.

## Introduction

*Neisseria meningitidis* is a human pathogen and a leading cause world-wide of fatal sepsis and meningitis, usually in otherwise healthy individuals [1]. The main reservoir of meningococcal carriage is the human nasopharynx, where occasionally this asymptomatic colonization is followed by invasive disease. The disease progression is rapid and much remains unknown about the disease process. Many meningococcal surface-associated virulence factors have been characterized; among these are pili, PilC, Opa, lipooligosaccharide (LOS) and the capsule. Meningococcal pili mediate attachment to host cells, and each pilus is composed of thousands of interactive PilE subunits, a pilus-associated adhesin PilC, and possibly other not yet identified components. Opa proteins are integral outer membrane proteins that mediate invasion of human cells [2]. Many surface exposed virulence factors of *Neisseria* undergo phase and antigenic variation, *i.e.* alteration of gene expression by on-off switching of a given gene product or by DNA rearrangement, which may augment bacterial adaptability and virulence [3], [4]. Opa expression undergoes phase variation due to frequent changes in the number of pentameric sequence repeats within the signal-peptide coding region, which alter the open-reading frame. Studies of bacterial virulence factors and host cell receptors have revealed complex and multifunctional contact points and signal transduction patterns during interaction between bacteria and target host cells [5]–[9]. Since *N. meningitidis* normally causes disease only in humans, most studies of bacteria-host cell interactions have been carried out *in vitro*, clearly limiting the understanding of disease progression.

Several experimental model systems using mice, rats and rabbits have been evaluated over the last few decades [10]. Iron-enhanced i.p. mouse infection utilizing a spectrum of iron sources clearly results in bacteremia [11], while i.n. infection in the presence of iron dextran also leads to colonization of nasal tissue [12]. However, the main disadvantage of these models is the unknown effect of iron on the host immune response and especially on the innate defense mechanisms. I.n. infection in infant mouse models may be used to study early events in the pathogenesis of meningococcal infection, however these models are not ideal since the bacteremia levels are low (maximum of 58%) while meningitis seldom develops [13], [14]. Infant rat models with i.p. bacterial challenge results in high bacterial counts in blood and CSF and high mortality rates [15], [16], but still requires the premature immune system of infant animals.

CD46 is a human cell surface complement regulator that interacts with *Neisseria* and several other microbes [17]. CD46 transgenic mice infected intraperitoneally (i.p.) with *N. meningitidis* are susceptible to meningococcal disease in contrast to nontransgenic mice that clear the infection and survive [18]. Importantly, in the CD46 transgenic mouse model, infection leads to sepsis and meningitis in adult mice without preinjection of enhancers, such as iron compounds. Bacterial replication in the blood and passage through the blood-brain barrier (BBB) occur in CD46 transgenic mice, but not in nontransgenic mice. Furthermore, induction of early immune responses after i.p. challenge of the transgenic mice resemble manifestations of meningococcal disease in humans [19]. Therefore, CD46 transgenic mice represent a suitable model system to study meningococcal disease and test vaccines *in vivo* in adult mice.

In this study, we used *in vivo* bioluminescence imaging (BLI) to monitor meningococcal sepsis in CD46 transgenic mice. Imaging over time demonstrated waves of bacterial growth and clearance, where bacterial signals diminished and then reappeared at novel locations. Reappearance of bacteria was associated with turning on of Opa expression. Furthermore, *N. meningitidis* accumulated in the thyroid gland and nasal cavity. Interestingly, the meningococcal adhesin PilC1 was dispensable for development of bacteremia and for bacterial translocation to the cerebrospinal fluid (CSF), but greatly enhanced bacterial translocation from blood to mucosal surfaces of the upper respiratory tract.

## Results

### *In vivo* imaging of bioluminescent *N. meningitidis*

**Figure 1:**
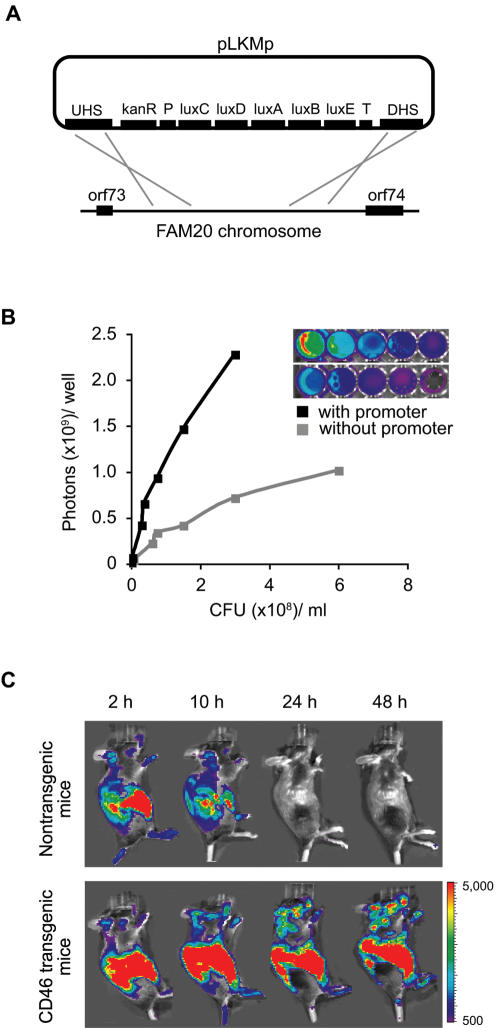
Generation and characterization of the bioluminescent *N. meningitidis* strain FAM20^LU^. (A) Chromosomal integration of a bioluminescence expression cassette into strain FAM20. The luxCDABE operon of plasmid pLKMp is flanked by the *Neisseria* specific *porA* promoter (P) and terminator (T) sequences of the *gapdh* gene. The kanamycin resistance gene, *kanR*, enables selection on agar-plates. UHS and DHS represent two sequences homologous to a non-coding region between orf73 and orf74 of the FAM20 genome. (B) The *porA* promoter enhances bioluminescent emission. Bioluminescence (in photon units) emitted from *N. meningitidis* strain FAM20 transformed with pLKMp (black line) or transformed with control vector without promoter (grey line) is plotted against bacterial counts. (C) *In vivo* BLI of mice challenged i.p. with 10^8^ FAM20^LU^. Mice were imaged at indicated time points post-infection. In contrast to nontransgenic mice (upper panel), strong signals in the body together with focal signals in the brain were detected in CD46 transgenic mice (lower panel). Images show one representative mouse from each group.

In order to image the progression of meningococcal disease *in vivo*, we constructed bioluminescently labeled *N. meningitidis*. The luxCDABE genes, which are required for the production of bioluminescent light, were expressed under the control of the meningococcal *porA* promoter and inserted into a non-coding chromosomal region of the meningococcal strain FAM20 (Figure 1A). As shown in Figure 1B, insertion of the *porA* promoter significantly increased the bioluminescent signal. The bioluminescent strain FAM20**^LU^** maintained expression of pili, PilC, LOS and the capsule, and showed no changes in the *pilE* gene sequence or growth rate as compared with the parent strain (data not shown). To evaluate the virulence of FAM20**^LU^**, we used a previously reported mouse model [18] and challenged CD46 transgenic mice i.p. with 10^8^ FAM20**^LU^**. After 24 h, strong bioluminescent signals were detected in the mouse body and in the brain of CD46 transgenic mice, but no signals were seen in nontransgenic mice (Figure 1C). As the original FAM20 strain, FAM20**^LU^** caused lethal disease within 2 days after infection. Further, the intensity of the emitted signals was proportional to the bacterial counts in blood and tissues (data not shown), allowing both temporal and spatial analysis regarding meningococcal disease progression. Construction of FAM20**^LU^** resulted in both Opa**^ON^** and Opa**^OFF^** clones, which showed no differences in growth, in adhesion and invasion of host pharyngeal epithelial cells, or in virulence using the CD46 transgenic mouse model (Figure S1 and data not shown). In summary, the data from both *in vivo* and *in vitro* experiments demonstrate that the novel bioluminescent strains are stable and have equivalent virulence as compared with the parental strain.

### Three diverse disease patterns of meningococcal disease in CD46 transgenic mice

**Figure 2:**
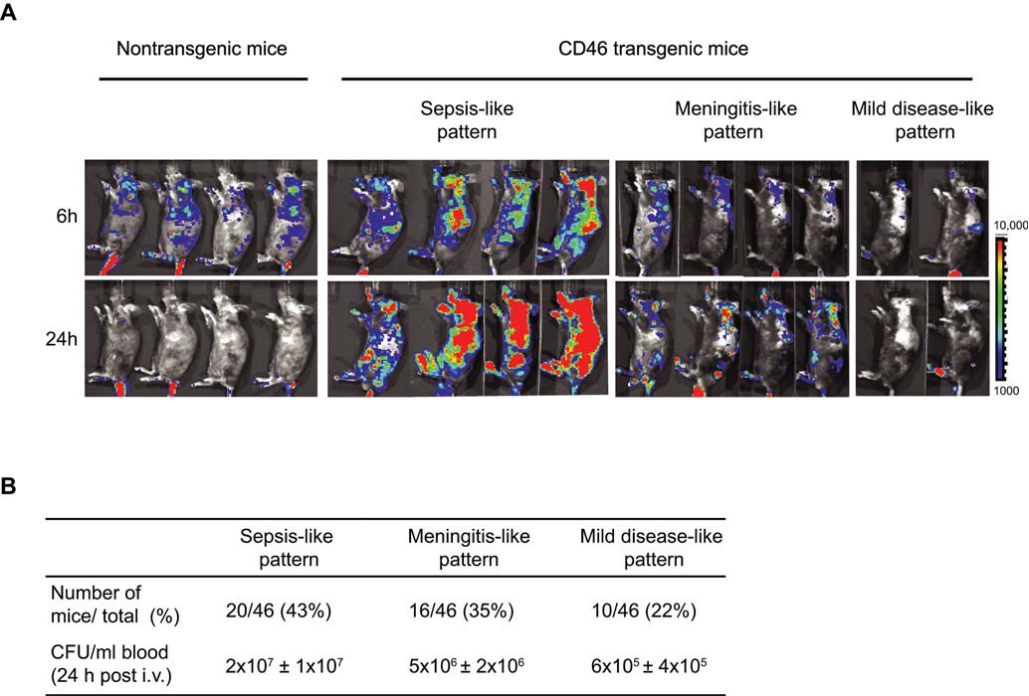
Meningococcal disease patterns in CD46 transgenic mice after i.v. infection. (A) BLI of nontransgenic mice and CD46 transgenic mice challenged i.v. with 10^9^ FAM20^LU^ at 6 and 24 h post-infection. Four mice are shown from each group, out of totally 46 mice. Signals at the tail region originate from the injection site. (B) Frequency of disease patterns and bacteremia levels (mean ± SD) in each group.

Meningococcal disease begins when bacteria successfully enter the bloodstream and survive host defenses. To study meningococcal sepsis we set up an intravenous (i.v.) infection model in CD46 transgenic mice. We found that 1×10^9^ bacteria were an optimal dose to induce severe meningococcal sepsis with a lethal outcome. Infection with fewer bacteria, *i.e.* 5×10^8^/mouse, resulted in clearance and 100% survival (data not shown). The optimal infection dose was high, however, recent reports have shown that patients with meningococcal disease may have bacterial loads of up to 6×10^8^ bacteria/ml blood [20], [21]. Infection with 10^9^ CFU/mouse corresponds to 5×10^8^ bacteria/ml in blood, which is within the range of bacterial load found in humans. Thus, mice were challenged with 10^9^ bacteria/mouse and analyzed by bioluminescent imaging at different time points. As shown in Figure 2A, nontransgenic mice infected with *N. meningitidis* cleared the bacteria within 24 h, while infection of CD46 transgenic mice resulted in three different disease patterns. Many of the CD46 transgenic mice, *i. e.* 43% (20/46), demonstrated a “sepsis-like” pattern resulting in a lethal outcome within 2 days after infection. In 35% (16/46) of the mice, a “meningitis-like” disease pattern was observed with signals mainly located in the brain and spinal region. Puncture of the cisterna magna of the mice in this group revealed high bacterial numbers in CSF 24 h post-infection (data not shown) and these mice died 2 or 3 days post challenge. Interestingly, a “mild disease-like” pattern appeared in a smaller number of mice (10/46), which displayed relatively low bacteremia levels of less than 10^6^ CFU/ml (Figure 2B). Thus, infection of CD46 transgenic mice leads to three different patterns of disease.

### Disease progression selects for Opa expression

**Figure 3:**
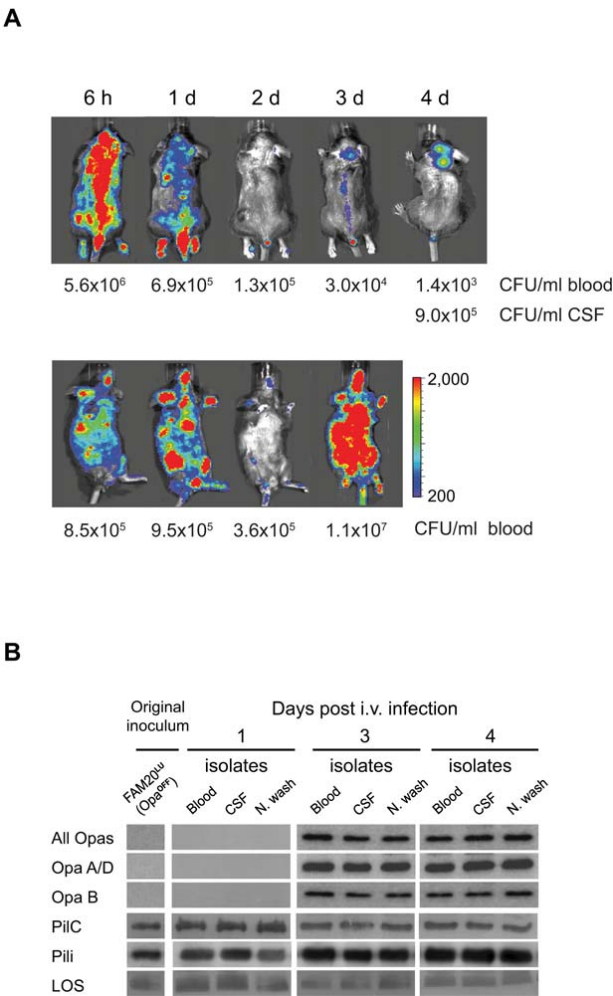
Dynamic meningococcal disease progression selects for Opa expression. (A) Mice with mild-disease pattern were imaged over 4 days using a detection scale of 200/2000 counts. Bioluminescent signals from the mice were undetectable at 2 days (2d) post-infection. From day 3 (3d), the signals reappeared either in the brain (upper panel) or in the whole body (lower panel). Images are representative of each subgroup. Each panel follows one mouse over time. Bacterial loads in blood and CSF at indicated time points post-challenge are shown. (B) *N. meningitidis* was isolated from blood, CSF or nasal washes (N wash) at day 1, 3 and 4 after infection. Recovered isolates together with the initial inoculum, were analyzed for expression of Opa proteins (30 kDa), PilC (110 kDa), and pili (PilE subunit 16 kDa) by immunoblots. LOS (6.5 kDa) was analyzed by Tricine SDS-PAGE.

We were interested to further analyze the mild meningococcal disease. To study disease dynamics a more sensitive detection scale was used to monitor bacterial load at 6 h, 1 day, 2 days, 3 days and 4 days after i.v. infection (Figure 3A). Unexpectedly, the signals disappeared after 2 days and the mice appeared to have recovered. However, the signals reappeared day 3. Five out of ten mice showed bacteria accumulating in the brain (Figure. 3A, top panel), while the bacterial counts in the CSF were almost 1000 times higher than in the blood. The remaining mice displayed strong signals in the whole body (Figure 3A, lower panel), with high bacterial blood counts, indicating fulminant bacteremia. Bacterial reappearance after the apparent recovery always led to a lethal outcome. These data imply that meningococcal dissemination *in vivo* is more complicated than we have previously understood. Reappearance of bacteria at day 3 and 4 suggested survival of a small population of bacteria. In an attempt to identify possible phase and antigenic variation of meningococcal virulence factors, bacteria were recovered from thyroid gland, thymus, lung, liver, kidney, spleen, intestine, nasal wash, CSF, and blood, at day 1, 3 and 4 after infection with FAM20**^LU^**Opa**^OFF^**. All recovered bacterial isolates retained expression of PilC, pili, LOS, and the capsule (Figure 3B and data not shown). Further, sequence variation of the *pilE* gene was not detected (data not shown). Interestingly, in 67% (4/6) of the mice all isolates recovered at day 3 or later expressed Opa, whereas at earlier infection stages Opa-expression was negative (Figure 3B, upper panels). Immunoblot analysis demonstrated expression of OpaA and OpaB in all isolates, while OpaD remained undetectable as in the initial inoculum (Figure 3B and data not shown). The expression pattern of Opa proteins was consistent after subculturing bacteria *in vitro*, indicating that Opa expression was selected in mice. In order to analyze the impact of Opa in disease development, CD46 transgenic mice were challenged i.v. with an Opa**^ON^** isolate recovered from blood. The bacterial blood counts were significantly higher (5.6×10^8^±5×10^8^ CFU/ml) at 24 h post infection compared with the initial inoculum (2×10^7^±1×10^7^ CFU/ml, Figure 2B). Thus, these data implicate that Opa proteins play an important role during disease progression.

### Meningococci accumulate in the thyroid gland after intravenous infection of CD46 transgenic mice

**Figure 4:**
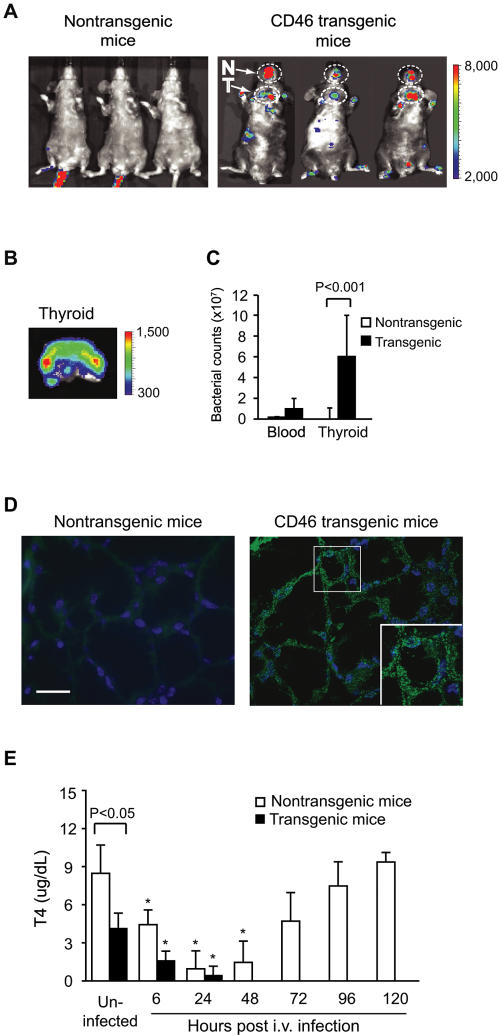
*N. meningitidis* targets the thyroid gland and the nasal area of CD46 transgenic mice. (A) BLI of mice at 24 h after i.v. infection. Distinct bioluminescent signals are visible in the thyroid area (T) and oral-nasal region (N) of CD46 transgenic mice. (B) *Ex vivo* BLI imaging of a dissected thyroid. (C) Bacterial loads in blood and thyroid at 24 h post-infection. Significant difference between thyroid of CD46 transgenic mice and nontransgenic mice is indicated (*P*<0.001). Bars indicate mean ± SD. (D) Immuno-histochemical examination of thyroid gland tissue sections of mice infected for 24 h. Tissue sections were overlaid with antiserum against *N. meningitidis*, followed by a FITC-conjugated anti-rabbit IgG. Cell nuclei were counter-stained with DAPI (blue). Bacteria (shown in green) are detected in the cellular layer of thyroid follicles of CD46 transgenic mice (inset). Scale bar: 20 µm. (E) Thyroxine (T4) levels in mice. Sera were collected from CD46 transgenic (n = 6) and nontransgenic mice (n = 6) at indicated time points post-infection and T4 levels were determined by enzyme immunoassay (EIA). Uninfected CD46 transgenic mice had significantly less T4 than nontransgenic mice (*P*<0.05). Significant reductions of T4 compared with uninfected mice are indicated with asterisks (*, *P*<0.05).

In addition to the bacterial signals in the mouse body and brain, *in vivo* bioluminescence imaging (BLI) also revealed strong signals that persisted over time in the thyroid gland and the nasal region of CD46 transgenic mice (Figure 4A). Further analysis of the thyroid gland demonstrated strong bioluminescence in a dissected thyroid (Figure 4B), and high bacterial counts in homogenized tissue (Figure 4C). Interestingly, the bacterial load was higher in the thyroid than in the blood. Immunohistochemical examination of thyroid tissue sections revealed that bacteria were primarily located in cellular layers, and not in the lumen of thyroid follicles (Figure 4D). Almost no bacteria were detected in thyroid glands from nontransgenic mice. In order to investigate the thyroid function during meningococcal disease, the blood level of the thyroid hormone, thyroxine (T4), was measured. Unexpectedly, uninfected CD46 transgenic mice exhibited lower T4 levels than nontransgenic mice (*P*<0.05). In response to infection, T4 levels dropped in both CD46 transgenic mice and nontransgenic mice. After 3 days of infection, T4 levels increased in nontransgenic mice as the mice recovered, whereas T4 levels were undetectable in CD46 transgenic mice after 2 days, which then had a lethal outcome (Figure 4E).

### *N. meningitidis* translocates from blood to the respiratory mucosa

**Figure 5:**
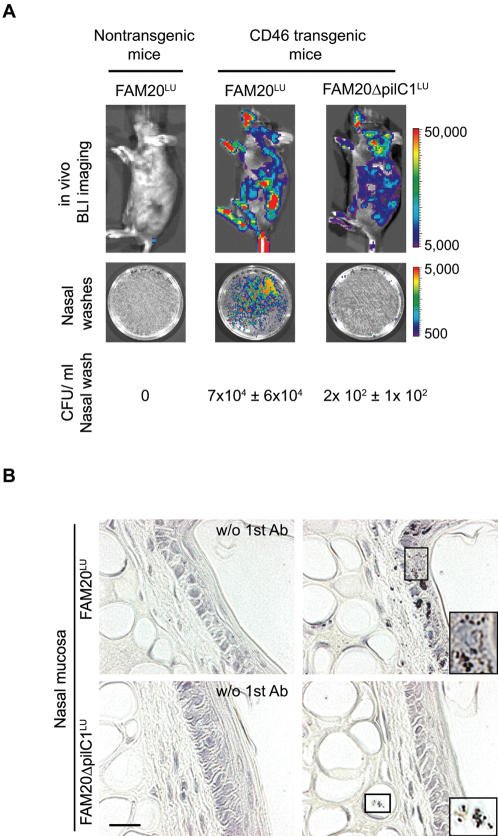
Translocation of *N. meningitidis* to the respiratory mucosa is dependent on PilC1. (A) Bioluminescent imaging of mice and nasal washes spread on GC-plates 24 h post infection. CD46 transgenic mice were infected i.v. with wild-type FAM20^LU^ or the PilC1-deficient mutant FAM20Δ*pilC1* ^LU^. Nontransgenic mice were challenged with FAM20^LU^. Bacterial counts in the nasal washes (CFU/ml) are presented. Significantly fewer PilC1 mutants were recovered compared with wild-type bacteria (*P*<0.05). (B) Immunohistochemical detection of *N. meningitidis* in nasal cavity sections of CD46 transgenic mice at 24 h post-infection. Bacteria (dark brown) were detected with rabbit anti-*N. meningitidis* antibody followed by HRP-conjugated goat anti-rabbit IgG secondary antibody, and stained with DAB. Control sections have been treated without the first antibody (w/o 1st Ab). Scale bar: 20 µm.

*In vivo* BLI also revealed a strong focal signal in the oral-nasopharyngeal region of infected CD46 transgenic mice (Figure 4A). This signal was detected over several days, sometimes with increasing intensity. To analyze the presence of bacteria in the nasopharynx, we collected nasal washes and measured the bacterial counts. At 24 h post-infection, high numbers of bacteria (7×10^4^ CFU/ml) were collected from nasal washes of CD46 transgenic mice, with over 95% of the recovered bacteria being bioluminescent meningococci (Figure 5A). No meningococci were found in nasal washes from infected nontransgenic mice or uninfected mice kept in the same cage as the challenged mice (Figure 5A and data not shown), indicating that meningococcal colonization of the respiratory mucosa is not a consequence of aerosol transfer of bacteria from other infected mice. Bacterial transmigration to the respiratory mucosa was further verified by immunohistochemical examination. Large numbers of meningococci were identified in the mucosa and submucosa of the nasal cavity of CD46 transgenic mice challenged with FAM20**^LU^** (Figure 5B, upper panel). Furthermore, clusters of meningococci were found in the nasal cavity and tracheal lumen, occasionally associated with the mucosal surface (data not shown). These data suggest the novel idea that *N. meningitidis* can translocate from blood to the respiratory mucosal surface in CD46 transgenic mice.

### Bacterial translocation from blood to the respiratory mucosa is PilC1 dependent

**Figure 6:**
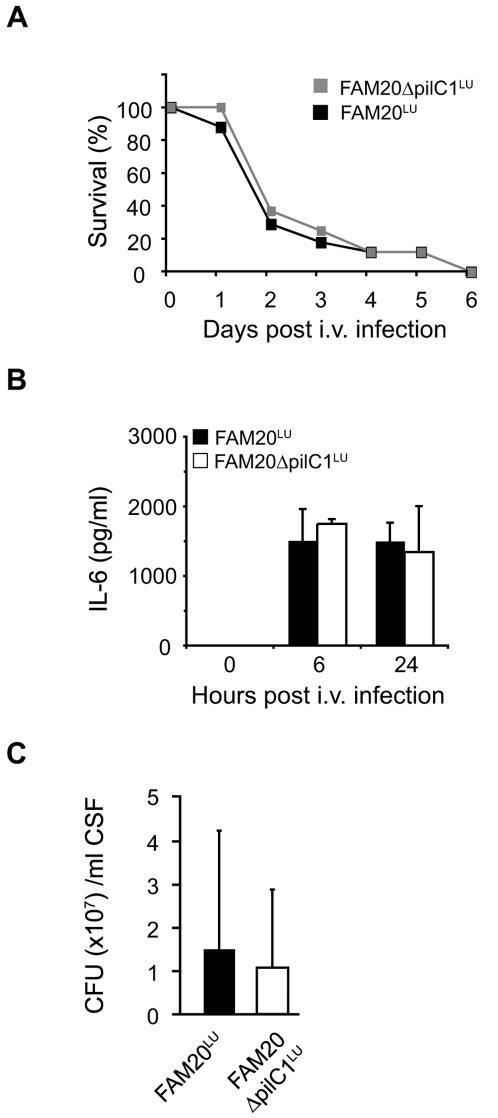
*In vivo* virulence properties of FAM20Δ*pilC1* ^LU^. (A) Survival rates of CD46 transgenic mice infected i.v. with PilC1-deficient FAM20Δ*pilC1* ^LU^ (grey line) or PilC-expressing FAM20^LU^ (black line). Survival was monitored for 6 days. (B) Induction of the proinflammatory cytokine, IL-6, in serum at 0 h, 6 h and 24 h post-infection was measured by ELISA. Data are presented as mean ± SD. (C) Bacterial counts in CSF of CD46 transgenic mice at 48 h after infection with FAM20Δ*pilC1* ^LU^ (open bars) or FAM20^LU^ (black bars). Data are presented as mean ± SD.

The pilus-associated adhesin PilC1 plays a critical role in mediating attachment to host epithelial target cells [22], [23]. In order to study the role of PilC1 in meningococcal disease and bacterial targeting to the respiratory mucosal surface, we generated a bioluminescently labeled PilC1 mutant. The resulting strain designated FAM20Δ*pilC1* **^LU^** displayed equivalent growth rates *in vitro* as compared to FAM20**^LU^** (data not shown). I.v. infection of mice with the bioluminescent PilC1 mutant, resulted in strong bacteremia, however, the bioluminescent signal in the nasal region was weak (Figure 5A). Nasal washes confirmed fewer meningococci in mice infected with the PilC1 mutant (2×10^2^ CFU/ml) than with the PilC1-expressing FAM20**^LU^** (7×10^4^ CFU/ml) (Figure 5A). Further, immunohistochemical examination failed to detect the PilC mutant in the epithelial cell layer, although minor amounts were detected in the submucosa (Figure 5B, lower panel). Surprisingly, the PilC1 mutant induced bacteremia, triggered IL-6, crossed the BBB, and caused lethal disease at similar rates as the wild-type (Figure 6). Taken together, *in vivo* investigations suggest that PilC1 is dispensable for bacteremia and further bacterial passage through the blood-brain barrier, but instead plays a crucial role in targeting *N. meningitidis* to the mucosa of the upper respiratory tract.

## Discussion

*Neisseria meningitidis* is a strict human specific pathogen that causes sepsis and meningitis. Meningococcal disease has a rapid onset with high mortality. The understanding of disease development is crucial for development of novel therapeutic strategies and vaccines against meningococcal disease. In this study, we used bioluminescent meningococci to image the disease progression in CD46 transgenic mice and demonstrated novel aspects of the dynamics of meningococcal disease. Imaging analysis of meningococcal sepsis revealed for the first time, three disease patterns, the two most common being sepsis and meningitis. The third group of mice developed a mild disease pattern during the first 2 days of infection. In humans, *N. meningitidis* is most commonly associated with rapid onset of meningitis and severe sepsis, but may also cause mild systemic meningococcal disease. Therefore, i.v. challenge of transgenic mice with *N. meningitidis* resembles human meningococcal disease features, supporting the system as a useful tool for studying meningococcal pathogenesis *in vivo*.

*In vivo* imaging and immunohistochemistry showed accumulation of bacteria in the thyroid gland of CD46 transgenic mice (Figure 4). This finding encouraged us to analyze the function of the thyroid by measuring the thyroid hormone T4 serum level. T4 was undetectable in CD46 transgenic mice after 2 days, whereas in nontransgenic mice T4 levels returned to normal as bacteria were cleared. These data indicate that an impaired thyroid function is associated with more severe disease. Uncontrolled release of proinflammatory cytokines occurs after meningococcal challenge of CD46 transgenic mice [19]. Competition for limited amounts of transcription factors involved in both cytokine synthesis and thyroid hormone metabolism could explain the resultant T4 reduction. In addition, it is also possible that decreased T4 levels inhibit the bactericidal capacity of polymorphonuclear leukocytes (PMNLs) since thyroid hormones can be an important iodine source for the myeloperoxidase-H_2_O_2_-halide antimicrobial and cytotoxic system in human PMNLs [24]. Interestingly, in a septic rat model thyroid hormone supplementation showed a beneficial effect on both survival and the severity of sepsis [25]. As shown in Figure 4E, CD46 transgenic mice have constitutively lower levels of T4 than the nontransgenic control mice. Further studies will address how CD46 influences the thyroid function and whether low constitutive T4 levels increases the susceptibility to meningococcal disease. It has been reported that T4 is reduced at the beginning of bacterial and viral meningitis in human infant patients [26]. However, there is no available data about the frequency of ongoing thyroid disorder in patients with meningococcal disease.

*N. meningitidis* can adapt to the host environment and escape the immune defenses via phase and antigenic variation of surface components. Earlier studies of experimental gonococcal urethritis in human volunteers have shown that Opa proteins are not required for the initial colonization of *N. gonorrhoeae* to host epithelium, instead Opa protein expression is selected during the course of infection [27]. In this study, *N. meningitidis* that reappeared in mice at later stages of infection invariably expressed Opa, independent of the isolation sites. The inoculum did not express detectable Opa protein. However, bacteria that translocated to CSF and respiratory mucosa at day 1 post-infection also lacked Opa, indicating that these steps are independent of Opa expression. Our data suggest that turning on Opa expression probably enhances late stage meningococcal adaptation and survival. Further, reappearance of bacteria shows that meningococci are capable of rapid growth in CD46 transgenic mice.

An interesting and novel finding in this study is the translocation of *N. meningitidis* to the respiratory mucosa of CD46 transgenic mice after i.v. infection. The PilC1 mutant did not reach the upper respiratory mucosa, indicating that bacterial interaction with respiratory epithelium is PilC1 dependent and probably not a result of vascular leakage in the nasal cavity. Thus, *in vivo* data in this study support previous *in vitro* findings that PilC1 mediates a direct interaction with epithelial cells [22]. Both PilC1-expressing *N. meningitidis* and the PilC1 mutant induced bacteremia, meningitis and uncontrolled release of proinflammatory cytokines. Therefore, PilC1 is most likely dispensable for meningococcal interactions with host immune defenses in blood and passage across the BBB. Since both the PilC1 mutant and the wild-type strain induced similar proinflammatory responses (Figure 6B), it is still possible that entry of the PilC1 mutant into the CSF may be a result from sepsis-induced cell damage of the BBB.

*N. meningitidis* reached the CSF and the respiratory mucosa in CD46 transgenic mice, but not in nontransgenic mice, suggesting that CD46 is a critical component of disease, either by interacting with bacteria at the site of translocation or by protecting bacteria at earlier stages allowing bacterial survival. Since the PilC1 mutant were not found at the respiratory mucosa, but crossed the BBB, it is likely that the mechanisms by which *N. meningitidis* interacts with these two cellular barriers are different. In humans, CD46 is expressed as four major isoforms, BC1, BC2, C1 and C2, depending on alternative splicing of an extracellular serine/threonine/proline rich domain and the choice between one of two cytoplasmic tails, Cyt-1 and Cyt-2 [28]. It has been reported that CD46 transgenic mice express distinct CD46 isoforms in throat epithelium and the BBB [18]. Hence, different mechanisms for bacterial adhesion and invasion might be required. Nevertheless, our data cannot exclude that an additional unknown factor may interact with PilC1 at the respiratory epithelium [29].

In conclusion, this study demonstrates that bioluminescent *N. meningitidis* strains together with the CD46 transgenic mouse model provide a potent tool for *in vivo* investigations of meningococcal disease. Disease dynamics and thyroid targeting during meningococcal sepsis revealed in this study might lead to new strategies to improve the clinical outcome in human patients.

## Materials and Methods

### Bacterial strains and growth conditions

*Escherichia coli* DH5α was used for cloning. *N. meningitidis* FAM20 belongs to serogroup C, MLST sequence type 11. The FAM20 PilC1 mutant strain, FAM20Δ*pilC1*, has been described previously [30]. *Neisseria* strains were grown at 37°C in a 5% CO_2_ atmosphere on GC agar (Difco) supplemented with Kelloggs, or in GC-liquid medium as described [19]. For selection of bioluminescent *N. meningitidis* transformants, kanamycin was added at concentrations of 50 µg/ml.

### Generation of bioluminescent *N. meningitidis* strains

Plasmid pXen-13 containing the luxCDABE operon was modified to pLKMp by insertion of a *Neisseria* specific promoter sequence, terminator sequence, kanamycin resistance gene, DNA uptake sequence and two DNA fragments homologous to a non-coding region of the FAM20 genome (Figure 1A and Table 1). The kanamycin resistance gene was PCR amplified from pZErO-2.1 (Invitrogen) and cloned into a *Sal*I/*Bam*HI site of the plasmid pXen-13. A 10-bp DNA uptake sequence required for efficient DNA transformation of *Neisseria* [31] was introduced into the 5′ end of the kanamycin cassette. Porin proteins are the most prevalent components of the *Neisseria* outer membrane and are essential for bacterial survival [32]. In order to increase the expression level of the luxCDABE operon in *N. meningitidis*, a 390 bp promoter sequence of the *porA* gene from strain FAM20 was PCR amplified and cloned into a *Bam*HI site upstream of the luxCDABE operon. Furthermore, a 200 bp transcription terminator region of the *gapdh* gene from strain FAM20 was amplified and cloned into a *Not*I site downstream of the luxCDABE operon. The plasmid contained the *porA* promoter upstream of the luxCDABE expression cassette was named pLKp. In addition, a plasmid without promoter was used as a control.

**Table 1:** Primers used for generation of the pLKMp plasmid

Product	Sequence of primer pairs*	Restriction site
KanR	5′-gatgaat**gtcgac**tactgggccgtctgaacaagggaaaacg-3′	*Sal* I
	5′-ctggaacaacac**ggatcc**ctatcgcggtctattcttttg-3′	*Bam*H I
Promoter	5′-aatagtac**gg atcc**gattca cttggtgctt cagcacc-3′	*Bam*H I
	5′-cgagggcggtaagtttttttcgc**agatct**gcttcc-3′	*Bgl*I I
Terminator	5′-ttcgcaggc***cggccg*** **taa**gg cttgaacaaa cctgtgg-3′	*Eae* I
	5′-tttgttcgcca**gcggccgc**aatatcaagttatagcgg-3′	*Not* I
UHS	5′-aacaatagagc**agtact**tcccggcaggtcaaattg-3′	*Sca* I
	5′-gctaacagaaaa**ctcgag**tccactattgttagggg-3′	*Xho* I
DHS	5′-cttggttaca**gcggccgc**atgattgcaaataatgag-3′	*Not* I
	5′-cgtaaatggtttca**gagctc**ataatttttctctttc-3′	*Sac* I

* Restriction enzyme cleavage sites are marked in bold. The DNA uptake sequence is underlined.

For chromosomal integration of plasmid pLKp by homologous recombination, two DNA fragments (UHS and DHS) from a region between orf73 and orf74 of the FAM20 genome, which does not contain inverted repeat sequences, were amplified and cloned into plasmid pLKp upstream of the kanamycin gene and downstream of the terminator, respectively. The luxCDABE expression cassette was integrated into the *N. meningitidis* strain FAM20 and the corresponding PilC1-deficient mutant. The transformation method has been described previously [33]. Single copy plasmid integration into the bacterial genome was confirmed by Southern blot analysis (data not shown).

### Mouse strains

The hCD46Ge transgenic mouse line was created using B6C3F1 hybrids. It harbors the complete human CD46 gene and expresses CD46 in a human-like pattern [34]. The F1 generation of C57BL/6 and C3H/Hen mice, *i. e.* B6C3F1, was therefore used as nontransgenic control in this work. All mice were bred at the animal facility of the Microbiology and Tumor Biology Center, Karolinska Institutet, and at the animal facility of the Biomedical Center, Uppsala University. Experiments were performed with 6–10 week old, age- and gender-matched mice and were repeated for five times with similar results. Animal care and experiments were in accordance with institutional guidelines and have been approved by national ethical committees.

### Animal procedures

*N. meningitidis* was suspended in GC medium to appropriate bacterial concentrations. Mice were infected i.p. or i.v. in the tail vein. CSF was withdrawn from cisterna magna and the CSF samples were checked for the absence of red blood cells before spreading on the GC agar. Nasal washes were collected as described previously [35]. Organs were excised from sacrificed mice, weighed and homogenized in PBS. Blood samples were obtained from the tail vein. Bacterial counts were determined by plating serial dilutions of samples on GC plates.

### Bioluminescence imaging

For *in vivo* imaging, mice were anaesthetized and imaged for a maximum of 2 min using an IVIS imaging system (Xenogen Corporation/Caliper Life Sciences) according to the manufacturer's instructions. Total bioluminescence emission from whole mouse or defined areas within the images of each mouse was quantified (in photon units) using the Living Image software package (Xenogen Corporation/Caliper Life Sciences). The dark fur of the mice was shaved away in order to increase the sensitivity of signal detection. For *in vitro* studies, images of bacterial plates or dissected organs were acquired with a 1 min exposure. The bioluminescence signals remained stable over time since bacteria recovered from mice produced similar signals as compared with the inoculum. Further, the bacterial blood counts in septic mice were proportional to the intensity of the bioluminescent signals over time.

### Immunoblotting, LOS preparation, capsule measurement, and DNA sequencing

Expression of meningococcal opacity (Opa) proteins, pilus-associated proteins (PilC), and type IV pili proteins was detected by immunoblot analysis. Sample preparation and immunoblot conditions have been described previously [36]. Opa-specific mouse monoclonal antibodies (H22.1, H21.1, 4B12/C11, and 7-24-D9) were kind gifts from Dr. M. Achtman (Max Planck Institut fuer Molekular Genetik, Berlin, Germany) and Dr. J. Cannon (University of North Carolina, United States). 4B12/C11 recognizes all Opa proteins, H21.1 recognizes OpaB, H22.1 recognizes OpaA and OpaD, and 7-24-D9 recognizes OpaD [37]. PilC antiserum was generated in rabbits against the full-length PilC1 of *N. meningitidis* FAM20. Pili antiserum was produced in rabbits against highly purified pili of *N. meningitidis* FAM20 [30]. LOS was isolated, separated on tricine SDS-PAGE and visualized by silver staining [33]. Capsule expression was determined by co-agglutination analysis. The *pilE* gene of 5 independent isolates recovered from blood, CSF and nasal washes at different time points after infection was amplified by PCR and sequenced using primers 5′-GATGCCGCAAATTTCCAATC-3′ and 5′-TCACGACCGGGTCAAACC-3′.

### Histology and immunohistochemistry

Mice were anesthetized and transcardially perfused with PBS. Organs were removed, fixed in 4% paraformaldehyde and embedded in paraffin. For detection of meningococci the tissue sections were incubated with rabbit antibody against *N. meningitidis* (diluted 1∶50; USBiological) for 60 min, horseradish peroxidase (HRP)-conjugated goat anti-rabbit IgG (diluted 1∶50; BioRad) for 60 min, and stained using ImmunoPure metal Enhanced DAB substrate (Pierce). For fluorescent immunohistochemistry, FITC-conjugated goat anti-rabbit IgG (diluted 1∶100; Santa Cruz) was applied as a secondary antibody. Sections were visualized with Carl Zeiss Axio Vision 2.05 image processing and analysis system (Zeiss).

### Determination of IL-6 and Thyroxine (T4) levels in serum

Concentrations of murine IL-6 and T4 in mouse serum was determined in duplicate using ELISA (Diaclone) and a Opticoat® Thyroxine (T4) enzyme immunoassay (EIA) kit (Biotecx Labs) according to manufacturer's recommendations.

### Statistical analysis

Differences in survival rates were assessed using a Fisher's exact test. Bacterial counts in blood and organs were analyzed by non-parametric Mann-Whitney test. A two-sided student's *t*-test was used to assess significance in the cytokine and thyroxine assays. Values of *P*<0.05 were considered significant.

## Supporting Information

Figure S1 Adhesion to and invasion into FaDu cells by the parent FAM20 and the bioluminescent FAM20^LU^ strains.
(0.24 MB DOC)
