# Supplementary material for: Imaging of Disease Dynamics during Meningococcal Sepsis
Source: PLoS One. 2007 Feb 21;2(2):e241. doi: 10.1371/journal.pone.0000241 (PMC1797199; doi:10.1371/journal.pone.0000241)
Supplement: Figure S1 — Adhesion to and invasion into FaDu cells by the parent FAM20 and the bioluminescent FAM20LU strains. (0.24 MB DOC) [file pone.0000241.s001.doc]

**Figure S1.** **Adhesion to and invasion into FaDu cells by the parent FAM20 and the bioluminescent FAM20^LU^ strains.** The nasopharyngeal epithelial cell line FaDu was grown in 24-well tissue culture plates (Costar) to full confluency. Cells were washed once before infection and suspensions of meningococci were added at a multiplicity of infection of 100. Binding to cells was allowed for 2 h at 37 °C in 5% CO_2_ atmosphere. Non-adhered bacteria were washed away and the infected cells were lysed with 1% saponin for 5 min. Bacteria were serially diluted and spread onto GC plates. The number of bacteria per cell was determined by counting colony-forming units after overnight incubation. To determine bacterial invasion, the cells were infected for 6 h, extracellular bacteria were then washed away, and 100 μg/ml gentamycin was added for 1 h to kill any remaining cell-associated bacteria before the saponin treatment. Bacterial adhesion or invasion was expressed as a comparison to the parent strain FAM20, which was set as 100%. Both assays were carried out in triplicate in three independent experiments. No significant differences were detected between the bioluminescent strains and the parent strain.
